# Natural Occurrence of 2′,5′-Linked Heteronucleotides in Marine Sponges

**DOI:** 10.3390/md8020235

**Published:** 2010-02-02

**Authors:** Annika Lopp, Tõnu Reintamm, Anne Kuusksalu, Indrek Tammiste, Arno Pihlak, Merike Kelve

**Affiliations:** Department of Gene Technology, Tallinn University of Technology, Akadeemia tee 15, 12618 Tallinn, Estonia; E-Mails: annika.lopp@ttu.ee (A.L.); tonu.reintamm@gmail.com (T.R.); anne.kuusksalu@ttu.ee (A.K.); indrek.tammiste@ttu.ee (I.T.); arpihlak@gmail.com (A.P.)

**Keywords:** 2′,5′-oligoadenylate synthetase, OAS, marine sponge, 2′,5′-linked heteronucleotides, natural products

## Abstract

2′,5′-oligoadenylate synthetases (OAS) as a component of mammalian interferon-induced antiviral enzymatic system catalyze the oligomerization of cellular ATP into 2′,5′-linked oligoadenylates (2-5A). Though vertebrate OASs have been characterized as 2′-nucleotidyl transferases under *in vitro* conditions, the natural occurrence of 2′,5′-oligonucleotides other than 2-5A has never been demonstrated. Here we have demonstrated that OASs from the marine sponges *Thenea muricata* and *Chondrilla nucula* are able to catalyze *in vivo* synthesis of 2-5A as well as the synthesis of a series 2′,5′-linked heteronucleotides which accompanied high levels of 2′,5′-diadenylates. In dephosphorylated perchloric acid extracts of the sponges, these heteronucleotides were identified as A2′p5′G, A2′ p5′U, A2′p5′C, G2′p5′A and G2′ p5′U. The natural occurrence of 2′-adenylated NAD^+^ was also detected. *In vitro* assays demonstrated that besides ATP, GTP was a good substrate for the sponge OAS, especially for OAS from *C. nucula*. Pyrimidine nucleotides UTP and CTP were also used as substrates for oligomerization, giving 2′,5′-linked homo-oligomers. These data refer to the substrate specificity of sponge OASs that is remarkably different from that of vertebrate OASs. Further studies of OASs from sponges may help to elucidate evolutionary and functional aspects of OASs as proteins of the nucleotidyltransferase family.

## 1. Introduction

2′,5′-oligoadenylate synthetases (OAS; EC 2.7.7.-) were discovered as a part of the interferon antiviral pathway in mammals [1]. When activated by double-stranded RNA, the enzymes catalyze the oligomerization of cellular ATP into 2′,5′-linked oligoadenylates (2-5A) with the general formula pppA(2′p5′A)_n_, where n ≥ 1. Currently, the only known function of 2-5A is to bind and activate a latent endoribonuclease (RNase L) that degrades single-stranded RNA of viral and cellular origin [2–4]. These enzymes together with 2′-phosphodiesterase which degrades 2-5A, constitute an RNA degradation pathway referred to as the 2-5A system [5]. In addition to functioning in the antiviral host defense, OAS as a component of 2-5A system has also been implicated in the regulation of other cellular processes, such as cell growth, differentiation and apoptosis [6–9]. Besides, biological roles for OAS proteins distinct from activation of the RNase L pathway have been suggested. Ghosh *et al.* have shown that a specific isozyme of OAS can induce apoptosis which is not mediated by the endoribonuclease [10]. OAS may regulate gene transcription and thereby have additional effects on cytokine receptor signal transduction pathways [11]. Physiological concentrations of 2-5A can be involved in the regulation of gene expression by virtue of a direct inhibition of DNA topoisomerase I [12].

Studies revealed that OAS is not only an ATP polymerase and should be defined as 2′-nucleotidyl transferase [13–15]. In the experiments with partially purified OAS preparations isolated from different sources (mammalian or chicken), the enzyme was able to catalyze *in vitro* transfer of the 5′-nucleotidyl moiety of a donor nucleoside triphosphate NTP (N is G, U, C, T, dA, dG, dC, dT) to a 2′-OH end of an acceptor molecule, such as ATP, NpA (3′,5′), NAD**^+^** or tRNA. The addition of a donor nucleoside triphosphate other than ATP terminates the chain elongation. The requirement for the acceptor site was defined as an AMP group linked in a RpA configuration, where R stands for pyrophosphate, NAD^+^, oligomeric or polymeric primers [15]. By using purified preparations of human OASs (69-kDa and 100-kDa isoforms), Marie *et al.* showed the capacity of the human enzyme to use GTP as an alternative substrate for OAS, as donor, and also as acceptor molecules [16]. However, GTP was a poor substrate compared with ATP; in the presence of ATP, guanylate moiety was transferred as donor on a 2-5A matrix followed by the release of the product [16]. Probably the best known natural 2′,5′-phosphodiester bond (between adenylate and guanylate moieties) is the one that forms in the lariat RNA intermediate during gene splicing [17]; as OAS can also be located in the nucleus, the role of OAS in generating that bond has been suggested [18,19]. Sperling *et al.* proposed that OAS activity might be involved in pre-mRNA splicing [18].

Despite the elucidated nucleotidyltransferase properties of OAS, the natural occurrence of 2′,5′-oligonucleotides other than 2-5A has never been demonstrated. Furthermore, the biological role of 2′,5′-linked heteronucleotides, which might be synthesized in cells, is not known [1,15,20,21].

Based on their structural motifs OAS proteins belong to the ancient group of nucleotidyltransferases, DNA polymerase β-like nucleotidyltransferase superfamily [22–24], being unique in catalyzing the formation of 2′,5′-phosphodiester linkage instead of the usual 3′,5′-linkage. Studies of OAS gene distribution among metazoans have shown their wide occurrence [25,26]; however, the genomes of several organisms, even of those belonging to certain vertebrate lineages (e.g., some teleost fishes and the amphibian *Xenopus tropicalis*) do not reveal their presence. The OAS genes have evidently been lost in some branches of the evolutionary tree of life [21,26–28].

Kuusksalu *et al.* have demonstrated the presence of OAS in the evolutionarily most basal multicellular animals, sponges [29,30]. Several cDNAs encoding oligoadenylate synthetases have been cloned from sponges [31–34]. Besides, a recombinant enzyme from *Geodia cydonium* was characterized for its OAS activity [35]. The constructed phylogenetic tree reveals that the known sponge OAS sequences fall into the same branch; vertebrate OASs are more distantly related [34]. Considering that the homology between primary structures of OAS from vertebrates and sponges is rather low and their respective genomic structures are different, sponge OASs may be classified as a distinct subgroup of OAS [36].

The role of OAS in sponges is still not known. The participation of this enzyme in responses to environmental stressors [37] or in host-defense reactions (including bacterial and viral infection) has been suggested [32,34]. However, the exact signalling pathways leading to any of these responses have not been elucidated. The genes that define the interferon response in mammals (including those for IFNs themselves) are absent from the known invertebrate genomes [38].

OAS activity can be detected in a variety of marine sponges (Demospongia), which exhibit different 2–5A synthesizing capacities and product profiles [39]. Compared with the enzymes of the mammalian OAS family, OASs from some sponge species (*G. cydonium*, *Chondrosia reniformis*, *Chondrilla nucula*) exhibit a surprisingly high activity [39]. The specific activity of OAS from these sponge crude extracts is comparable or greater than that of the IFN-stimulated human (HeLa) or murine (L929) cells [37,40]. In addition, the natural occurrence of 2-5A, in the amounts that were characteristic of IFN-stimulated virus-infected cells, has been found in *G. cydonium* [29,37].

This study shows that two other marine sponges, *Thenea muricata* and *Chondrilla nucula*, can accumulate 2-5A in high amounts. We demonstrate that besides 2-5A these sponges are capable of synthesizing *in vivo* a series of 2′,5′-linked heteronucleotides, in which all four ribonucleotides (ATP, GTP, UTP, CTP) are included. After dephosphorylation, they appeared in the form of A2′p5′N. NAD^+^ could be also used as *in vivo* substrate for OAS, since 2′-adenylated derivative of NAD^+^ was found in the extract of *T. muricata*. In addition to ATP and NAD^+^, GTP was used *in vivo* as an acceptor molecule for oligomerization to produce G2′p5′N (N is A or U). Reactions performed under *in vitro* conditions with these substrates confirmed the results obtained *in vivo*.

Interestingly, our present study of the substrate specificity revealed that the sponge OAS is also able to catalyze (2′–5′) oligomerization of pyrimidine nucleotides. Therefore, as a result of nucleotidyltransferase activity of the sponge OAS, which is considerably different from that of vertebrate enzymes, a variety of 2′,5′-linked oligonucleotides can be obtained under *in vitro* conditions.

In the light of the new data obtained in the study we suggest that closer studies of OASs from sponges, the most basal multicellular animals, would help us to elucidate original function(s) of the enzymes of this nucleotidyltransferase family. On the other hand, such data could be useful for understanding the functions of mammalian OASs that remain outside the scope of RNase L.

## Results and Discussion

### 2.1. Analysis of sponge perchloric acid extracts

**Figure 1:**
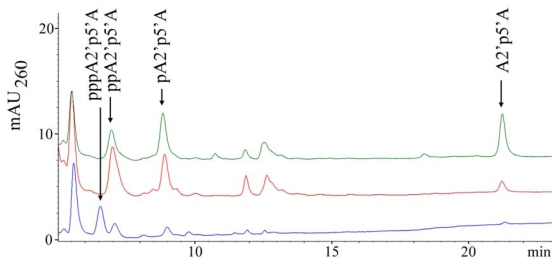
The occurrence of 2′,5′-diadenylates in the form of tri-, di- and monophosphates, and “core” forms in perchloric acid extracts of *T. muricata*. HPLC analysis was performed by applying gradient 1 (see Experimental section). HPLC chromatograms correspond to approximately 6 mg of the sponge wet weight. Blue, red and green colored lines represent samples which were cultivated in laboratory tanks for different periods of time.

**Figure 2:**
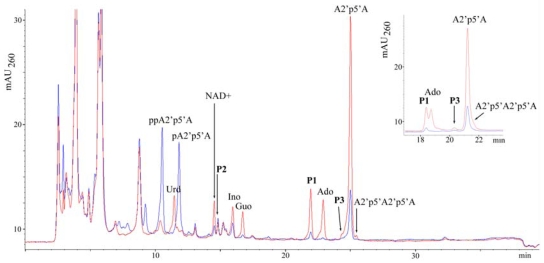
The occurrence of 2′,5′-linked nucleotides in a perchloric acid extract of *T. muricata*. HPLC analysis was performed by applying gradient 2 (see Experimental section). HPLC chromatogram corresponds to approximately 8 mg of the sponge wet weight. Blue line represents the nucleotide profile of a perchloric acid extract of the sample, red line–the product profile of the same sample after dephosphorylation of the extract. *Inset*: the analysis of the sample by applying HPLC gradient 1. The peaks P1-P3 were collected and analyzed: P1 involves A2′p5′G, P2–NAD2′p5′A, P3–A2′p5′U and A2′p5′C.

The HPLC method for analyzing cellular nucleotides after their extraction from animal cells or tissues is widely used for quantification of adenine nucleotides. In the course of such a study for which a series of samples of the sponge *T. muricata* were used we found that this sponge contains considerable amounts of diadenylates with 2′,5′-phosphodiester linkage. These diadenylates were present in the perchloric acid extract in various 5′-phosphorylated (tri-, di-, mono-) and unphosphorylated (“core”) forms, the proportions of which depended on the particular sample (Figure 1). In some samples minor amounts of 2′,5′-triadenylates were also present (Figure 2). Such data indicate that the oligoadenylates, initially synthesized as 5′-triphosphate derivatives resulting from OAS activity exhibited by the sponge were afterwards dephosphorylated to a different degree. However, the mechanisms that regulate phosphatase activities in particular sponges are not known yet.

Treatment of the extract with alkaline phosphatase simplified the subsequent analysis as all compounds were separated in their “core” forms (Figure 2). Quantification of adenylates revealed that their amounts varied by an order of magnitude between different samples of *T. muricata* (see Experimental section, 3.1): the amount of 2′,5′-linked diadenylates (A2′p5′A) was 2–50 pmol/mg wet weight, whereas that of adenosine (reflecting the total amount of adenine nucleotides ATP, ADP and AMP) varied from 2 to 80 pmol/mg wet weight.

Surprisingly, in many samples 2-5A was present in quantities that considerably exceeded those of the total adenine nucleotide pool (Figure 2). Such a variation between the amounts of adenylates obviously shows differences in the physiological status of sponges resulting i) from cultivation effects and ii) from variability between different specimens. In accordance with the data published previously [37], elevated levels of 2-5A evidently correspond to stress conditions for the sponge (data not shown).

**Figure 3:**
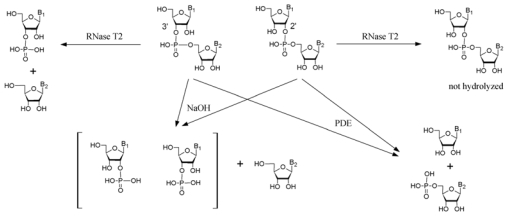
Determination of the primary structure and proof of 2′,5′-linkage in an oligoribonucleotide. Alkaline hydrolysis of an oligomeric nucleoside produces 2′- and 3′-monophosphates while the 3′-terminal base is found as a nucleoside. Digestion with snake venom phosphodiesterase produces 5′-monophosphates while the 5′-terminal base is found as a nucleoside. Ribonuclease T2 hydrolyzes any 3′,5′-internucleotide linkage between ribonucleotides, but oligomers with 2′,5′-internucleotide linkage are resistant to this nuclease.

A peak with retention time close to adenosine and UV spectrum (λ_max_ = 256 nm) different from oligoadenylates regularly accompanied high levels of A2′p5′A. Its intensity increased after dephosphorylation of the extract and we presupposed the presence of some hetero-oligomer, which might also have a 2′,5′-linkage. Therefore, the chromatographic fraction containing the unknown compound (peak 1, Figure 2) was collected and subjected to respective chemical and enzymatic treatments (see Figure 3) to establish the structure of the compound. Since alkaline hydrolysis of this compound produced equimolar amounts of the products [(3′-AMP + 2′-AMP) and guanosine)], and digestion with snake venom phosphodiesterase (PDE) produced equimolar amounts of 5′-GMP and adenosine, the unknown compound has to be a (A, G) dimer. Resistance to the treatment with ribonuclease T2 indicated that the dimer has a 2′,5′-internucleotide linkage instead of the usual 3′,5′-linkage.

**Figure 4:**
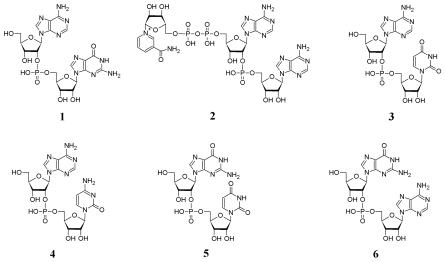
Structures of compounds **1**–**6**.

As a result of these treatments, the unknown compound was identified as adenylyl(2′-5′)guanosine (A2′p5′G) (Figure 4, **1**). Subsequent mass-spectrometric analysis (m/z 611.2) confirmed the identity of the product.

**Figure 5:**
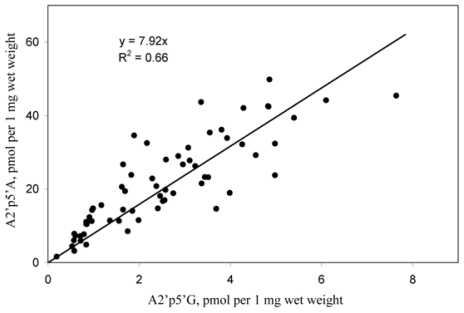
A correlation between the amounts of A2′p5′A and A2′p5′G in different samples (n = 64) of *T. muricata*. Dephosphorylated perchloric acid extracts of *T. muricata* were analyzed by HPLC. The amounts of 2′,5′-linked compounds were calculated from the same HPLC runs.

The mean content of A2′p5′G in different samples was about a magnitude lower than that of A2′p5′A′ A positive correlation observed between the amounts of A2′p5′A and A2′p5′G in different samples of *T. muricata* (Figure 5) indicated that formation of the (A, G) dimer was directly related to the activity of OAS in a particular sponge sample. Such a finding inspired us to look more carefully at minor components present in different samples in order to search for additional oligonucleotides with 2′,5′-linkage.

The area of a peak next to NAD^+^ (peak 2, Figure 2) correlated positively with the A2′p5′A peak as well (not shown), but differently from A2′p5′A, A2′p5′G and NAD^+^ peak, its area did not increase by phosphatase treatment of the extract. The UV spectrum of that peak was very similar to NAD^+^. Such a behavior (including the observed retention time) would be expected for Nir-5′pp5′-Ado-2′p5′-Ado (NAD2′p5′A), a compound formed from NAD^+^ and ATP by mammalian OAS [15]. MALDI-MS analysis of this compound in positive ion mode was in agreement with NADpA (m/z 993.1; 992 Da for neutral molecule). However, since NAD^+^ molecule has two ribose moieties with free 3′ and 2′ OH groups, 5′-AMP (from the substrate ATP) might bind, alternatively, to nicotinamide ribose moiety. The negative ion mode MALDI-MS analysis proved to be useful in solving this problem. The analysis of NAD^+^ in negative ion mode yielded the strongest signals with m/z 540 (the ionized molecule lacking nicotinamide) and 426 (corresponds to the fragment of ADP), whereas the peak of expected mass (m/z 662) was low. Appearance of NAD^+^ in negative ion mode MALDI-MS analysis as fragments with m/z 540 > 426 has been reported, while the signal with m/z 662 has not been detected at all [41]. Similarly to NAD^+^, NADpA in negative ion mode yielded the strongest signals with m/z 869.1 and 755.1, together with the low intensity signal that corresponded to the expected mass with m/z 991.1. On the basis of the fragments formed in both cases we can conclude that the adenylate additional to NAD^+^ in NADpA was bound to adenine nucleotide moiety of the NAD^+^ molecule. Since NADpA isolated from the sponge extract was resistant to RNase T2 treatment, the identified compound was Nir-5′pp5′-Ado-2′p5′-Ado (Figure 4, **2**).

NAD^+^ has been reported to be a preferred acceptor molecule for the OAS in human (HeLa) cell extracts, however, the respective 2′-adenylated derivatives of NAD^+^ were not detected in the extracts synthesizing 2-5A [42].

Another small peak in the samples with higher A2′p5′A content was found whose area increased after treatment of the extract with alkaline phosphatase (peak 3, Figure 2, inset). As a result of the respective treatments (Figure 3) and mass-spectrometric analysis, two compounds in the collected chromatographic fraction were identified: one of them was adenylyl(2′–5′)uridine (A2′p5′U) (Figures 4, **3**) (m/z 572.1) and the other was adenylyl(2′–5′)cytidine (Figure 4, **4**) (A2′p5′C) (m/z 571.1) (Figure 4). From these two compounds A2′p5′U was prevalent, its molar amount was about two orders of magnitudes lower than that of A2′p5′A.

Thus, dimers containing all four ribonucleotides in the form of A2′p5′N (N is A, G, U or C) were found in dephosphorylated extracts of *T. muricata*, the molar content of these compounds decreasing in the order: A2′p5′A > A2′p5′G > A2′p5′U > A2′p5′C. Although the exact meaning of the synthesis of such 2′,5′-linked oligomers for the sponge physiology is not known, decrease in cellular nucleotide content, especially that of ATP, would have negative impact on total metabolic activity and could indicate stress conditions for the sponge.

**Figure 6:**
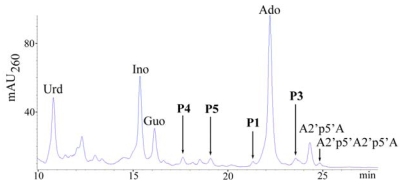
The occurrence of 2′,5′-linked compounds in a perchloric acid extract of *C. nucula*. HPLC analysis of a dephosphorylated sponge extract was performed by applying gradient 2 (see Experimental section). HPLC chromatogram corresponds to approximately 30 mg of the sponge wet weight. The peaks P1, P3, P4 and P5 were collected and analyzed: P1 involves A2′p5′G, P3-A2′p5′U and A2′p5′C, P4–G2′p5′U and P5–G2′p5′A.

To expand the knowledge about the natural occurrence of 2′,5′-linked heteronucleotides in sponges, the detectable levels of these metabolites could be found, guided by the example of *T. muricata*, in sponges with high 2-5A content. The occurrence of 2-5A has been shown only for *G. cydonium*, using radioimmunoassay method for establishing its concentration [29]. The content of 2-5A in *G. cydonium* that was determined in the present study was up to 5 pmol per mg wet weight, being in the same range with values found earlier. Two other sponges that had previously demonstrated high OAS activities [39] were studied for their 2-5A content. In *C. reniformis*, the amount of 2-5A was about 2 pmol per mg wet weight, whereas in *C. nucula* 2-5A was accumulated in higher amounts (14 and 3 pmol per mg wet weight for dimer and trimer, respectively) (Figure 6). Therefore, *C. nucula* was chosen for comparative studies of the occurrence of 2′,5′-linked heteronucleotides.

The presence of A2′p5′G (peak 1, Figure 6) was easily detected in dephosphorylated extracts of *C. nucula* and also confirmed as described above. Similarly to *T. muricata*, the amount of A2′p5′G (which also increased by phosphatase treatment) in the extract of *C. nucula* was about a magnitude lower than that of A2′p5′A.

To establish other possible 2′,5′-linked oligomers in the extract of *C. nucula*, chromatographic fractions (18 fractions, á 0.5 mL) in the region between guanosine and (2′–5′)ApApA peaks (Figure 6) were collected and analyzed. The following hetero-dimers (migrating together with some other components of the fraction) were identified in the analyzed region: peak 3 (23.5–24 min)–A2′p5′U (Figure 4, **3**) (m/z 572.1) and A2′p5′C (Figure 4, **4**) (m/z 571.1), together with traces of adenosine and A2′p5′A; peak 4 (17.5–18min)–guanylyl(2′–5′)uridine (G2′p5′U) (Figure 4, **5**) (m/z 588.1) as a minor component migrating together with dGuo; peak 5 (19–19.5 min)–guanylyl(2′-5′)adenosine (G2′p5′A) (Figure 4, **6**) (m/z 611.1) as a minor component migrating together with an unknown compound (UV_max_ 261 nm). The molar content of 2′,5′-linked dimers in the extract that was calculated by peak areas and from the data of alkaline hydrolysis of the collected fractions decreased in the order: A2′p5′A > A2′p5′U > A2′p5′G > A2′p5′C > G2′p5′U ≥ G2′p5′A.

In comparison with *T. muricata*, two additional compounds with the formula G2′p5′N (N is A or U) were found in the extract of *C. nucula*. In order to synthesize such hetero-oligomers, GTP has to be bound at acceptor site and ATP at donor site of the OAS. Since ATP is a prevalent ribonucleotide in cells (see also Figure 6 for the content of respective nucleosides), the affinity of GTP towards OAS from *C. nucula* should be considerable.

To date, no data are available in the literature about the ability of a sponge OAS to catalyze the formation of co-oligomers of ATP and another ribonucleotide. Therefore, to verify the hypothesis of *in vivo* synthesis of the 2′,5′-heteronucleotides in sponges, *in vitro* reactions were performed with NAD^+^ and four ribonucleotides, used alone or in mixtures with ATP.

### 2.2. The formation of 2′,5′-linked oligomers in vitro

The extracts with specific activities of 138 pmol ATP/min per μg protein and 1.64 nmol ATP/min per μg protein were used in the assays for *T. muricata* and *C. nucula*, respectively. NAD^+^ alone remained intact in the extract, however, other ribonucleotides which were used alone as substrates instead of ATP were easily converted to their respective 2′,5′-linked oligomers. In the case of GTP, the specific activity was 43 pmol/min per μg protein for *T. muricata* and 2.34 nmol/min per μg protein for *C. nucula*. Unexpectedly, pyrimidine nucleotides UTP or CTP were utilized quite efficiently to produce the respective 2′,5′-linked oligomers. The calculated specific activity for *T. muricata* was 54 pmol UTP/min per μg protein or 38 pmol CTP/min per μg protein. For *C. nucula*, the specific activities relative to pyrimidine nucleotides were 69 pmol UTP/min per μg protein and 13 pmol CTP/min per μg protein.

These data show that the specific activity of the ribonucleotides towards OAS decreases in the order: ATP > GTP ≅ UTP ≅ CTP or GTP ≅ ATP ≫ UTP > CTP for *T. muricata* and *C. nucula*, respectively.

The product profiles formed from the ribonucleotides were studied under the assay conditions where most of the given substrate was utilized for oligomerization. In these conditions, 2′,5′-linked oligomers up to pentamers (ATP), tetramers (GTP) and trimers (UTP and CTP) were formed (Table 1).

The relative amounts of the formed oligomers are presented in Table 2. As it can be seen, purine nucleotides were better substrates for chain elongation than pyrimidine nucleotides. The quantitative data of oligomerization of CTP by the *C. nucula* extract are not included in Table 2 due to a deaminating activity which was present in the extract. This resulted in a quite extensive conversion of CTP to UTP (with a specific activity of 22.6 pmol/min per μg protein) and in the production of a mixture of 2′,5′-linked products consisting of C and U homo-oligomers as well as of (C, U) heterooligomers.

Thus, as illustrated by the example of *T. muricata* and *C. nucula*, the substrate specificity of OAS depends on the particular sponge species. The inter-species comparison of known sponge OAS protein sequences refers to a very low similarity between these proteins, most probably due to their independent evolution after the divergence from their common ancestor [36]. Although the OAS genes from *T. muricata* and *C. nucula* have not been sequenced, different affinities of ribonucleotides towards OAS might be explained by differences in the enzyme structures of the respective sponges.

To study the formation of hetero-oligomers, ATP was combined with GTP, UTP, CTP or NAD^+^ in roughly equimolar concentrations. Since G2′p5′U was identified after phosphatase treatment of perchloric acid extract of *C. nucula*, the combination of GTP and UTP was used additionally in case of this sponge. Taking into account the relative ability of a sponge OAS to catalyze the oligomerization of these substrates when used alone (see above), the nature and multiplicity of the products formed by one or another of the sponges in the experiments with the mentioned mixtures is not surprising (Table 3).

**Figure 7:**
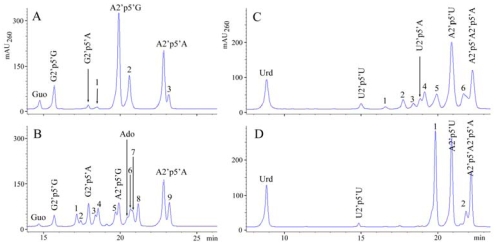
The oligomerization of ATP in the presence of GTP (A, B) or UTP (C, D) by OAS from the extract of *T. muricata* (A, C) or *C. nucula* (B, D) under *in vitro* conditions. Substrates were used in the following concentrations: 0.86 mM ATP, 0.67 mM GTP, 0.76 mM UTP. Incubation time was 1 h for *C. nucula* and 17 h for *T. muricata*. HPLC analysis of dephosphorylated reaction mixtures was performed by applying gradient 1 (see Experimental section). The chromatographic peaks were collected and analyzed. The numbered compounds in panels are: **A**–1 (G2′p5′G2′p5′G + G2′p5′A2′p5′A), 2 (A2′p5′A2′p5′G), 3 (A2′p5′A2′p5′A); **B**–1 (G2′p5′G2′p5′A), 2 (G2′p5′G2′p5′A2′p5′A), 3 (G2′p5′G2′p5′G), 4 (G2′p5′A2′p5′A), 5 (A2′p5′G2′p5′G), 6 (A2′p5′A2′p5′G), 7 (G2′p5′A2′p5′G), 8 (A2′p5′G2′p5′A), 9 (A2′p5′A2′p5′A); **C**–1 (m/z 1207′1, the sequence of nucleotides not analyzed), 2 (U2′p5′A2′p5′U), 3 (U2′p5′A2′p5′A2′p5′A), 4 (U2′p5′A2′p5′A), 5 (A2′p5′A2′p5′U + A2′p5′A2′p5′A2′p5′U), 6 (A2′p5′A2′p5′A2′p5′A + A2′p5′A); **D–**1 (A2′p5′ ′A2′p5′U), 2 (A2′p5′A).

The results demonstrate that adenylate was preferred in 5′-terminal position in hetero-oligomers, and the yield of dimeric product A2′p5′N was generally the highest (Figure 7A, C, D). As an exception, there was only a slight preference for ATP in 5′-terminal position when the ATP + GTP mixture was added to the *C. nucula* extract (Figure 7 B). In the reaction with GTP + UTP, GTP was an efficient acceptor molecule while UTP terminated the chain (Table 3). In the case of *T. muricata*, where the difference between OAS specific activities towards purine and pyrimidine nucleotides was smaller than that observed for *C. nucula*, pyrimidine nucleotides could also serve (though with a smaller efficiency) as acceptor molecules for oligomerization of ATP in the presence of UTP or CTP (Table 3, Figure 7C).

Thus, these data provide a clear proof for the ability of OAS to catalyze *in vivo* the production of 2′,5′-heteronucleotides. The results show that besides ATP, other nucleotides are efficiently used for the formation of 2′,5′-linked oligomers under *in vitro* conditions. Especially, the usage of pyrimidine nucleotides as acceptor molecules for the oligomerization refers to the substrate specificity of the sponge OAS that is remarkably different from that of vertebrate OASs.

It has previously been proposed that OAS proteins have evolved from a common ancestor of archaeal CCA-adding enzymes and poly(A) polymerases [24,25]. The OAS proteins show the highest similarity with the class I CCA-adding enzymes, which not only catalyze the synthesis of the CCAsequence to the 3′-tRNA end, but are also capable of poly(C) synthesis [43]. Interestingly, OASs from *T. muricata* and *C. nucula* are capable of catalyzing the synthesis of 2′,5′-linked homo-oligomers not only from ATP, but also from GTP, UTP and CTP. These findings indicate that OASs from sponges have broader nucleotide specificity than that known for vertebrate OASs. Further studies of these enzymes from sponges allow for better characterization of their nucleotidyltransferase properties.

Moreover, research in this field may help to understand the function of OAS proteins in sponges, the most basal multicellular animals. Taking into consideration that several OAS genes are expressed in a sponge and that, on the other hand, OAS protein sequences in different sponge species differ considerably from one another [36], the functions of OASs in sponges may also be diversified. Though at present suggestions about the role(s) of OASs in sponges are speculative, one can consider a possible role of these enzymes in the control of cell growth, which may be regulated by different signal pathways depending on the inducing factor. Another intriguing aspect is the participation of OASs in immune responses of sponges against bacteria or viruses as also proposed earlier [32,34,36]. The elucidation of the pathways involved in these host-defence reactions would give us valuable information about the innate immune system of invertebrates. In its turn, thorough studies of OASs from sponges could enlighten some aspects of the suggested multifunctionality of OAS proteins in vertebrates.

## 3. Experimental Section

### 3.1. Sponges

The marine sponges *Thenea muricata* (Demospongiae) were collected on a soft bottom sediment off Korsfjord (close to Bergen, Norway) at 300 m depths (60° 07′ 44 N, 004° 49′ 9 E). The sponges were either fixed directly in liquid nitrogen or maintained in tanks with running sea water for establishing a laboratory-based culture and then fixed. The total number of analyzed samples (corresponding to sponge specimens) was 64. The samples were kindly provided by Hans Tore Rapp from Bergen University. The marine sponges *Chondrilla nucula*, *Geodia cydonium* and *Chondrosia reniformis* (Demospongiae) collected from the Tyrrhenian Sea (Napoli Bay) were kindly provided by Dr. S. de Rosa (CNR, Napoli, Italy). The specimens of *G. cydonium* collected from the Adriatic Sea (Rovinj, Croatia) and *C. reniformis* collected from Cala Montgo, L’Escala (Spain) were gifts from Prof. Dr. W. E. G. Müller (University of Mainz, Germany) and Dr. M.-L. Schläppy (MPI for Marine Microbiology, Bremen, Germany), respectively. The animals were cut into pieces, frozen in liquid nitrogen and kept at −70 °C until their further use.

### 3.2. Sample homogenization

The sponge tissue was disrupted by means of TissueLyser (Qiagen, Retsch GmbH, Germany). Grinding jars were pre-chilled in liquid nitrogen and the frozen samples were pulverized in a highspeed regime (at 30 Hz) for 1 min.

### 3.3. Preparation of extracts

#### 3.3.1. Perchloric acid extract

0.5 mL of an ice-cold 0.6 M HClO_4_ solution was added per about 100–150 mg of a powdered frozen sample and mixed vigorously. The extract was additionally vortexed and kept on ice for 10 min. After centrifugation for 5 min (10,000 g, 4 °C), the supernatant was removed and neutralized with 5 N KOH to about pH 7. The KClO_4_ precipitate formed was removed by centrifugation (5 min, 10,000 g, 4 °C), the supernatant was collected and submitted to HPLC analysis either directly or after treatment with shrimp alkaline phosphatase (SAP, see *3.6*). In order to identify minor components present in the extracts, the extract was first concentrated (10–30 fold) by lyophilization under reduced pressure (SpeedVac Concentrator, Savant) and then analyzed, either directly or after treatment with SAP, by the HPLC method.

#### 3.3.2. Protein extract

The powdered frozen sponge tissue was extracted for 15 min at 0 °C with 10 mM HEPES (pH 7.5) buffer containing 100 mM KCl. The insoluble pellet was removed by centrifugation (5 min, 10,000 g, 4 °C) and the supernatant was used for activity assays. The protein concentration of the extract was measured by a modified Bradford method [44]. The extracts with protein concentrations of 2.5 mg/mL and 2.8 mg/mL were used for *T. muricata* and *C. nucula*, respectively.

### 3.4. 2′,5′-Oligoadenylate synthetase activity assays

The reaction mixtures containing 20 mM Tris-HCl, pH 8, 50 mM KCl, 5 mM MgCl_2_, the sponge protein extract (final protein concentration from 0.005 to 0.1 mg/mL) and the substrate(s) (ATP, GTP, UTP, CTP, NAD^+^) were incubated at 37 °C for required periods of time (5 min–20 h). The reaction was stopped by heating (4 min, 95 °C). Substrates were used in the following concentrations: 0.86 mM ATP, 0.67 mM GTP, 0.76 mM UTP, 0.78 mM CTP and 0.97 mM NAD^+^.

For the determination of specific activity where the appearance of the firstly synthesized dimer was registered, short reaction times (5–15 min) and low sponge protein concentrations (0.05 to 0.005 mg protein/mL) were suitable. To study the total pool of oligomeric products formed in different reactions, the protein concentrations were 0.1 mg/mL and incubation times were longer. Overnight incubations (17–20 h) for all substrates were suitable for *T. muricata*. For *C. nucula*, 1 h incubations were used with all substrates or their combinations, except for pyrimidine nucleotides UTP or CTP when used alone. In the latter case, the incubation time was 18 h.

Products of the reactions were analyzed either directly or after dephosphorylation of the reaction mixture (see 3.6).

### 3.5. HPLC analysis

The analysis was performed with an Agilent 1100 HPLC instrument (Agilent Technologies, USA) on a C_18_ reverse-phase column (Phenomenex Luna C18(2), 250 × 4.6 mm, 5 μm; pre-colum SecurityGuard C18, 4 × 3 mm). Eluent A was 50 mM ammonium phosphate pH 7.0 and eluent B was 50% methanol in water. The products were separated at 30 °C in a linear gradient of eluent B (0–40%, 20 min); the column was equilibriated with eluent A before the next injection (10 min) (gradient 1). Alternatively, the column was thermostated at 20 °C and the linear gradient of eluent B (0–60%, 30 min) was used (gradient 2). The absorption was measured at 260 nm. The adenylates and other products were identified by comparing their retention times with those of authentic compounds and by their UV spectra (using previously created UV spectra library). The retention times of the respective compounds with 2′,5′- and 3′,5′-linkage differed substantially, the latter having longer retention in the column′ For example, by applying gradient 1 the retention time of G2′p5′U was 16 min, whereas that of G3′p5′U (Sigma) was 20 min; the retention time of A2′p5′U was 20′9 min *versus* 23 min for A3′p5′U (Sigma).

The products were quantified by integrating the corresponding peak areas (software of Agilent Technologies). Taking into account the molar absorption coefficients of the compounds at 260 nm, molar concentrations were calculated. Molar absorption coefficients for 2′,5′-adenylates have been given previously [45]. The following molar absorption coefficients were used for other oligomers: G2′p5′G–2.11 × 10^4^, G2′p5′G2′p5′G–3.16 × 10^4^, G2′p5′G2′p5′G2′p5′G–4.21 × 10^4^, U2′p5′U–1.8 × 10^4^, U2′p5′U2′p5′U–2.7 × 10^4^, C2′p5′C–1.36 × 10^4^, C2′p5′C2′p5′C–2.04 × 10^4^.

### 3.6. Analysis of the composition of oligoribonucleotides by means of chemical and enzymatic hydrolysis

The composition of oligoribonucleotides was determined by analysis of the core oligomers after removal of the 5′-phosphate groups by alkaline phosphatase treatment. The dephosphorylation reaction was performed by adding the appropriate amounts of 10 × reaction buffer (100 mM MgCl_2_, 200 mM Tris-HCl, pH 8) and shrimp alkaline phosphatase (SAP, USB Corporation) at final concentration of 0.02–0.04 U/μL and incubating the mixture at 37 °C for 1 h; thereafter the enzyme was inactivated (5 min, 95 °C).

#### 3.6.1. Alkaline hydrolysis

Oligomer(s) of the collected chromatographic fractions were treated for 10 min at 95 °C with 0.3 M NaOH, placed on ice and neutralized to about pH 7 by adding the appropriate amount of 2 N HCl. The products of hydrolysis were identified by the HPLC method. The length of an oligomer was evaluated by means of quantification of the resulting products of hydrolysis.

#### 3.6.2. Snake venom phosphodiesterase treatment

Oligomers were treated in 50 mM Tris-buffer, pH 9 for 1 h at 37 °C with snake venom phosphodiesterase (phosphodiesterase from *Vipera lebetina* venom was kindly provided by Dr. J Siigur, NICBP, Tallinn) at final concentration of 0.04 mg/mL. The enzyme was inactivated by heating (5 min, 95 °C). Partial digestion of an oligomer by using shorter incubation times (5–30 min) and lower enzyme concentrations (0.004–0.008 mg/mL) allowed us to determine the nucleotide composition on the basis of resulting shorter fragments of the oligomer. The products were analyzed by the HPLC method.

#### 3.6.3. Ribonuclease T2 treatment

The oligomers under investigation were incubated overnight in 50 mM sodium acetate buffer, pH 4, at 35 °C with RNase T2 (Invitrogen) at final concentration of 0.01 U/μL. Reference oligoribonucleosides A3′p5′A (Sigma), A3′p5′A3′p5′A (BioSpring, Germany), A3′p5′U (Sigma), G3′p5′U (Sigma) were incubated for 1 h in the same conditions; all oligomers were totally degraded. The products of hydrolysis were identified by the HPLC method.

### 3.7. MALDI-MS analysis

The collected HPLC fractions were directly subjected to mass-spectrometric analysis. The analysis was carried out with a Bruker Daltonics Autoflex II TOF/TOF instrument in a reflector negative mode. THAP (2,4,6-trihydroxyacetophenone) matrix solution was used as described previously [39]. [THAP-H^+^], [ApA-H^+^], [ApApA-H^+^] and [ApApApA-H^+^] with m/z of 167, 595.14, 924.1 and 1253.15, respectively, were used as external calibrants. DHB (2,5-dihydroxybenzoic acid) matrix solution was used for registration of masses in the region with m/z from 547 to 549. The mass signals of NAD^+^ and NAD2′p5′A were registered also in a reflector positive mode, using THAP as a matrix solution. The discrepancies between registered and theoretical monoisotope masses were in the range of 0.1 Da.

## 4. Conclusions

The perchloric acid extracts prepared from the marine sponges *T. muricata* and *C. nucula* reveal the occurrence of 2′,5′-linked oligoadenylates, mostly in dimeric forms. As established by the example of *T. muricata*, concomitantly with accumulation of 2′,5′-diadenylates, several 2′,5′-linked heterodimers were also detected in the extracts. In particular, a positive correlation observed between the amounts of adenylyl(2′–5′)adenosine and adenylyl(2′–5′)guanosine (or NAD2′p5′A) refers to the formation of 2′,5′-linked heterodimers due to the activity of OAS in a particular sponge sample. After dephosphorylation of perchloric acid extracts, these 2′,5′-heterodimers were identified as A2′p5′N, where N is G, U or C (*C. nucula*, *T. muricata*); as NAD2′p5′A (*T. muricata*) and as G2′p5′N, where N is A or U (*C. nucula*). The case of G2′p5′U gives evidence for the presence of non-adenylate 2′,5′-linked compound in sponge cells as a result of OAS activity. Thus, in addition to ATP and NAD^+^, GTP was used *in vivo* as an acceptor for (2′–5′) oligomerization. Evidently, the formation of a particular 2′,5′-linked product by OAS depends on the characteristics of the enzyme and, on the other hand, on bioavailability of its substrates in sponge cells.

The *in vitro* assays demonstrated that in *T. muricata* ATP was a preferred acceptor for the OAS, while in *C. nucula* GTP was comparable with ATP as an acceptor for the enzyme. That can explain the natural occurrence of 2′,5′-linked heteronucleotides in *C. nucula*, which were identified in the form of G2′p5′A and G2′p5′U. Besides, the usage of pyrimidine nucleotides UTP or CTP as substrates *in vitro* was quite unexpected, since (2′–5′) oligomerization of pyrimidine nucleotides by vertebrate OASs has never been demonstrated. These results show that OASs from most basal multicellular animals, sponges, have substrate specificity properties that are substantially different from those of vertebrate enzymes. With OASs from the marine sponges *T. muricata* and *C. nucula*, a variety of 2′,5′-heterooligomers (up to tetramers) was obtained *in vitro* wherein all four ribonucleotides can serve as either acceptor or as donor substrates for oligomerization. Notably, the substrate specificity of an OAS depends on the particular sponge species, which might be explained by differences in the enzyme structures of the respective sponges. Taking into account a great number of extant marine sponge species, one could expect a much greater variability in the substrate specificity properties of sponge OASs in general. Thus, a future finding of new naturally occurring 2′,5′-heteronucleotides in sponges would be expected. On the other hand, the specific features of OASs from marine sponges open perspectives for enzymatic synthesis of authentic samples of a variety of 2′,5′-linked heteronucleotides.

## Figures and Tables

**Table 1:** Analytical forms of 2′,5′-linked homo-oligoribonucleotides.

Substrate	(2′–5′) Oligomer*^1^	Retention time, min*^2^	m/z_exp_*^3^

ATP	ApA	22.2	595.1
	ApApA	22.5	924.1
	ApApApA	21.9	1253.1
	ApApApApA	21.0	1582.3

GTP	GpG	14.9	627.1
	GpGpG	17.5	972.1
	GpGpGpG	17.9	1317.3

UTP	UpU	15.1	549.0
	UpUpU	16.3	855.1

CTP	CpC	10.6	547.0
	CpCpC	11.4	852.0

*^1^ the products were analyzed in their dephosphorylated forms.

*^2^ retention times correspond to the applied gradient 1 (see Experimental Section).

*^3^ experimentally obtained mass signal in negative ion mode MALDI-MS.

**Table 2:** The relative molar amounts of 2′,5′-linked homo-oligomers (%) formed from different ribonucleotides.

	*Thenea muricata*					
Substrate	Incubation time, h	Monomer*	Dimer	Trimer	Tetramer	Pentamer

ATP	17	3.4	27.6	48.7	18.5	1.9
GTP	20	5.7	67	24.1	3.3	-
UTP	20	5.1	85.6	9.3	-	-
CTP	20	7.4	88.1	4.5	-	-

	*Chondrilla nucula*					

ATP	1	4.1	31.3	59.6	5	-
GTP	1	10.1	50.6	35.7	3.6	-
UTP	18	13.6	76.4	10	-	-

* –the amount of the respective mono-nucleotide remaining at the end of incubation.

**Table 3:** 2′,5′-linked heteronucleotides formed *in vitro* from the substrates added to the extract of the sponge *Thenea muricata* (1) or *Chondrilla nucula* (2).

Substrates	(2′–5′) Oligomer*^1^	Retention time, min*^2^	m/z_exp_*^3^	Sponge

ATP + GTP	GpGpA	16.7	956.1	2
	GpGpApA	16.9	1285.2	2
	GpA	17.5	611.1	1, 2
	GpApA	18.1	940.1	1, 2
	ApGpG	19.2	956.1	2
	ApG	19.4	611.1	1, 2
	ApApG	20.2	940.2	1, 2
	GpApG	20.3	956.2	2
	ApGpA	20.7	940.2	2

ATP + UTP	UpApU	17.7	878.0	1
	UpApApA	18.4	1230.1	1
	UpA	18.8	572.0	1
	UpApA	19.1	901.0	1
	ApApU	19.9	901.0	1, 2
	ApApApU	19.9	1230.1	1
	ApU	20.9	572.0	1, 2

ATP + CTP	CpApC	13.7	876.1	1
	CpA	15.5	571.1	1
	CpApA	15.5	900.1	1
	ApApApC	19.5	1229.2	1
	ApApU*^4^	19.9	901.1	2
	ApApC	19.9	900.1	1, 2
	ApU*^4^	20.9	572.1	2
	ApC	21.0	571.1	1, 2

GTP + UTP	GpGpU	14.6	933.1	2
	GpGpGpU	15.7	1278.1	2
	GpU	16.0	588.1	2

ATP + NAD^+^	NADpA	13.2	993.1*^5^	1, 2

*^1^,**^2^,***^3^ –see explanations of Table 1.

*^4^ –these products were formed due to a deaminating activity of the extract of *C. nucula* which resulted in a partial conversion of CTP to UTP (see above).

*^5^ –mass signal in positive ion mode MALDI-MS.
